# Supplementary material for: Large-scale exome analyses reveal new rare variant contributions in amyotrophic lateral sclerosis
Source: Nat Genet. 2026 Mar 31;58(4):717–25. doi: 10.1038/s41588-026-02535-9 (PMC13083253; doi:10.1038/s41588-026-02535-9)
Supplement: Supplementary file 1 — Supplementary Tables 1 and 2, Figs. 1–12 and Consortium lists. [file 41588_2026_2535_MOESM1_ESM.pdf]

nature genetics

Supplementary information https://doi.org/10.1038/s41588-026-02535-9

Large-scale exome analyses reveal new rare variant contributions in amyotrophic lateral sclerosis

In the format provided by the authors and unedited

Supplementary Tables 2
Supplementary Figures 4
Consortium Authors 18

Supplementary Tables

Supplementary Table 1 | Variant effect predictions for variants identified in either the exome-wide single variant analysis or the targeted single variant analysis among GCEP-curated genes. D: damaging, B: benign, P: possibly damaging.

	Variant	Gene	Consequence	Effect	CADD	Polyphen2HDIV	Polyphen2HVAR	MutationTaster	SIFT	REVEL	AlphaMissense
exome-wide discovery	9:133154147:C:A	GBGT1	c.455G>T/p.R152L	missense_variant	0.192			D	D	0.18567	
	7:44206388:A:G	YKT6	c.191A>G/p.Y64C	missense_variant	31.000	D	D	D	D	0.91371	0.5892 (likely_pathogenic)
	1:223762207:A:G	CAPN2	c.1588A>G/p.I530V	missense_variant	19.310	B	B	D	B	0.19193	0.0949 (likely_benign)
	3:184057041:A:G	HTR3C	c.556A>G/p.T186A	missense_variant	17.710	P	P	B	D	0.70276	0.1091 (likely_benign)
	12:122547457:T:A	KNTC1	c.859T>A/p.W287R	missense_variant	25.300	D	D	D	B	0.69696	0.8073 (likely_pathogenic)
GCEP	21:31667359:T:C	SOD1	c.341T>C/p.I114T	missense_variant	27.400	D	D	D	D	0.99931	0.2771 (likely_benign)
	21:44333234:C:A	CFAP410	c.172G>T/p.V58L	missense_variant	10.340	B	B	B	B	0.04649	0.1784 (likely_benign)
	21:31667290:A:C	SOD1	c.272A>C/p.D91A	missense_variant	14.850	B	B	D	B	0.81659	0.1494 (likely_benign)
	4:169424668:G:C	NEK1	c.3107C>G/p.S1036*	stop_gained	36.000			D		.	
	21:31659783:C:T	SOD1	c.14C>T/p.A5V	missense_variant	31.000	D	D	D	D	0.95470	0.8401 (likely_pathogenic)
	4:169585374:C:T	NEK1	c.782G>A/p.R261H	missense_variant	28.200	D	D	D	D	0.57090	0.6029 (likely_pathogenic)
	12:57581917:C:T	KIF5A	c.2957C>T/p.P986L	missense_variant	20.500	B	B	D	D	0.37995	0.0745 (likely_benign)
	12:64488537:T:C	TBK1	c.1391T>C/p.V464A	missense_variant	22.100	B	B	D	B	0.30369	0.1748 (likely_benign)
	1:11022464:A:G	TARDBP	c.1055A>G/p.N352S	missense_variant	18.270	B	B	D	B	0.59130	0.0523 (likely_benign)
	3:35738257:C:T	ARPP21	c.1688C>T/p.P563L	missense_variant	26.700	D	D	D	B	0.59375	
	16:31191418:C:T	FUS	c.1561C>T/p.R521C	missense_variant	25.300	B	B	D	D	0.86636	0.9881 (likely_pathogenic)
	12:64481900:A:G	TBK1	c.871A>G/p.K291E	missense_variant	27.900	D	D	D	D	0.74657	0.3415 (ambiguous)
	1:11022556:A:G	TARDBP	c.1147A>G/p.I383V	missense_variant	16.460	B	B	D	B	0.69527	0.0722 (likely_benign)
	3:35792484:C:T	ARPP21	c.2240C>T/p.P747L	missense_variant	24.000	D	B	D	D	0.13305	0.1727 (likely_benign)
	10:80170859:C:T	ANXA11	c.112G>A/p.G38R	missense_variant	24.200	D	D	D	D	0.56562	0.9022 (likely_pathogenic)
	4:169580841:C:G	NEK1	c.868+1G>C	splice_donor_variant&intron_variant	32.000			D		.	
	16:31191431:C:T	FUS	c.1574C>T/p.P525L	missense_variant	23.700	P	B	D	D	0.90764	0.9952 (likely_pathogenic)
	X:56565398:C:T	UBQLN2	c.1525C>T/p.P509S	missense_variant	18.920	B	B	B	B	0.75143	0.2195 (likely_benign)

Supplementary Table 2 | Representative Gene Ontology (GO) terms for the candidate genes. Terms were summarized using the *rrvgo* R package, where terms were clustered by semantic similarity and a representative term was selected for each cluster based on its uniqueness score. *A full list of terms is available in Supplementary Data 11.

Gene symbol	GO BP	GO MF	GO CC
UNC13C	• protein-containing complex organization<br>• neurotransmitter transport<br>• synaptic transmission, glutamatergic<br>• dense core granule exocytosis<br>• synaptic vesicle docking	• calmodulin binding<br>• calcium ion binding<br>• diacylglycerol binding<br>• syntaxin-1 binding	• neuromuscular junction<br>• vesicle membrane<br>• plasma membrane region<br>• neuron projection
KIF4A	• chromosome organization<br>• intracellular transport<br>• microtubule-based movement<br>• spindle midzone assembly<br>• cell division	• isomerase activity<br>• metal cluster binding<br>• microtubule motor activity<br>• cytoskeletal protein binding	• nuclear matrix<br>• midbody<br>• neuron projection<br>• microtubule associated complex<br>• cytoplasmic region<br>• supramolecular complex
TTC3	• proteolysis<br>• modification-dependent macromolecule catabolic process	• aminoacyltransferase activity	• nucleolus<br>• Golgi apparatus
HTR3C	• ligand-gated ion channel signaling pathway<br>• nervous system process<br>• regulation of postsynaptic membrane potential<br>• synaptic signaling<br>• transmembrane transport<br>• response to oxygen-containing compound	• serotonin receptor activity<br>• transporter activity	• neuron projection<br>• synapse<br>• serotonin receptor complex<br>• transporter complex<br>• plasma membrane region
YKT6	• membrane organization<br>• vesicle localization<br>• membrane docking	• SNAP receptor activity<br>• cell adhesion molecule binding<br>• S-acyltransferase activity	• mitochondrion<br>• cell body<br>• neuron projection<br>• SNARE complex<br>• vesicle membrane<br>• Golgi membrane<br>• apical dendrite<br>• endoplasmic reticulum-Golgi intermediate compartment membrane
KNTC1	• cell division<br>• organelle assembly<br>• protein-containing complex organization<br>• protein localization to organelle<br>• kinetochore assembly<br>• exit from mitosis<br>• organelle localization	• GTPase binding	• actin cytoskeleton<br>• kinetochore microtubule
GBGT1	• glycosylation<br>• carbohydrate metabolic process<br>• lipid glycosylation<br>• liposaccharide metabolic process	• acetylgalactosaminyltransferase activity	• Golgi membrane
CAPN2*	• reproductive process<br>• positive regulation of developmental process<br>• multi-multicellular organism process<br>• behavior<br>• interleukin-6 production<br>• cellular response to stress<br>• cellular response to interferon-beta<br>• positive regulation of cellular component organization	• protein-containing complex binding<br>• cytoskeletal protein binding<br>• calcium ion binding<br>• calcium-dependent cysteine-type endopeptidase activity	• vacuole<br>• membrane microdomain<br>• Golgi apparatus<br>• cell surface<br>• cell body<br>• chromatin<br>• pseudopodium<br>• perinuclear region of cytoplasm

Supplementary Figures

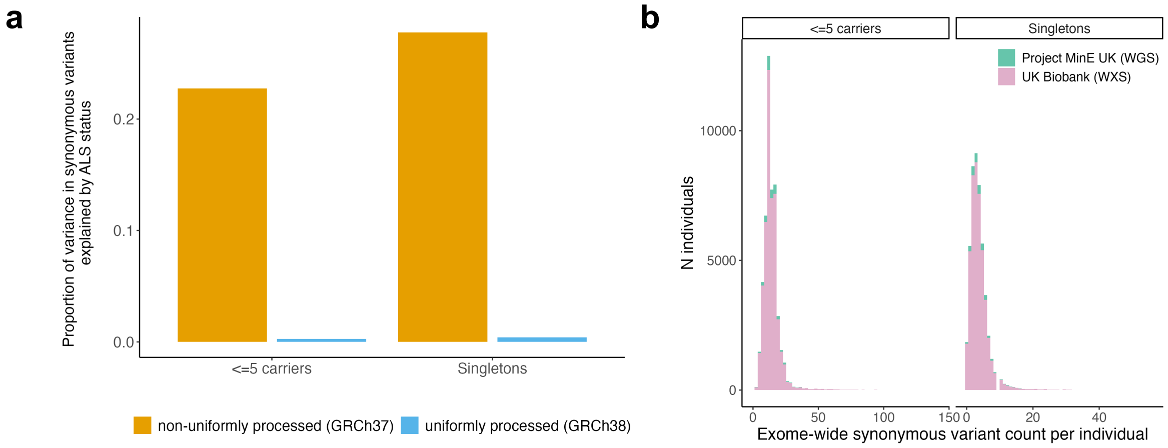

Supplementary Figure 1 | Uniform reprocessing of sequencing datasets overcomes systematic technical biases. a, Proportion of variance explained (R^2) of total number of synonymous variants per individual by ALS status among 28,582 samples that were either uniformly processed and aligned to GRCh38 or non-uniformly processed and aligned to GRCh37 respectively. It shows that the proportion of variance explained by total synonymous variant count was substantially reduced by uniformly processing the sequencing data. **b,** Distribution of the total number of ultra-rare (≤5 carriers; left) and singleton-only (right) synonymous variants among two ancestry-matched cohorts in the uniformly processed (GRCh38) callset (WGS: Project MinE UK, WXS: UK Biobank). The distributions of exome-wide ultra-rare counts are aligned between these two cohorts, indicating that sequencing technologies are comparable after joint processing and quality control.

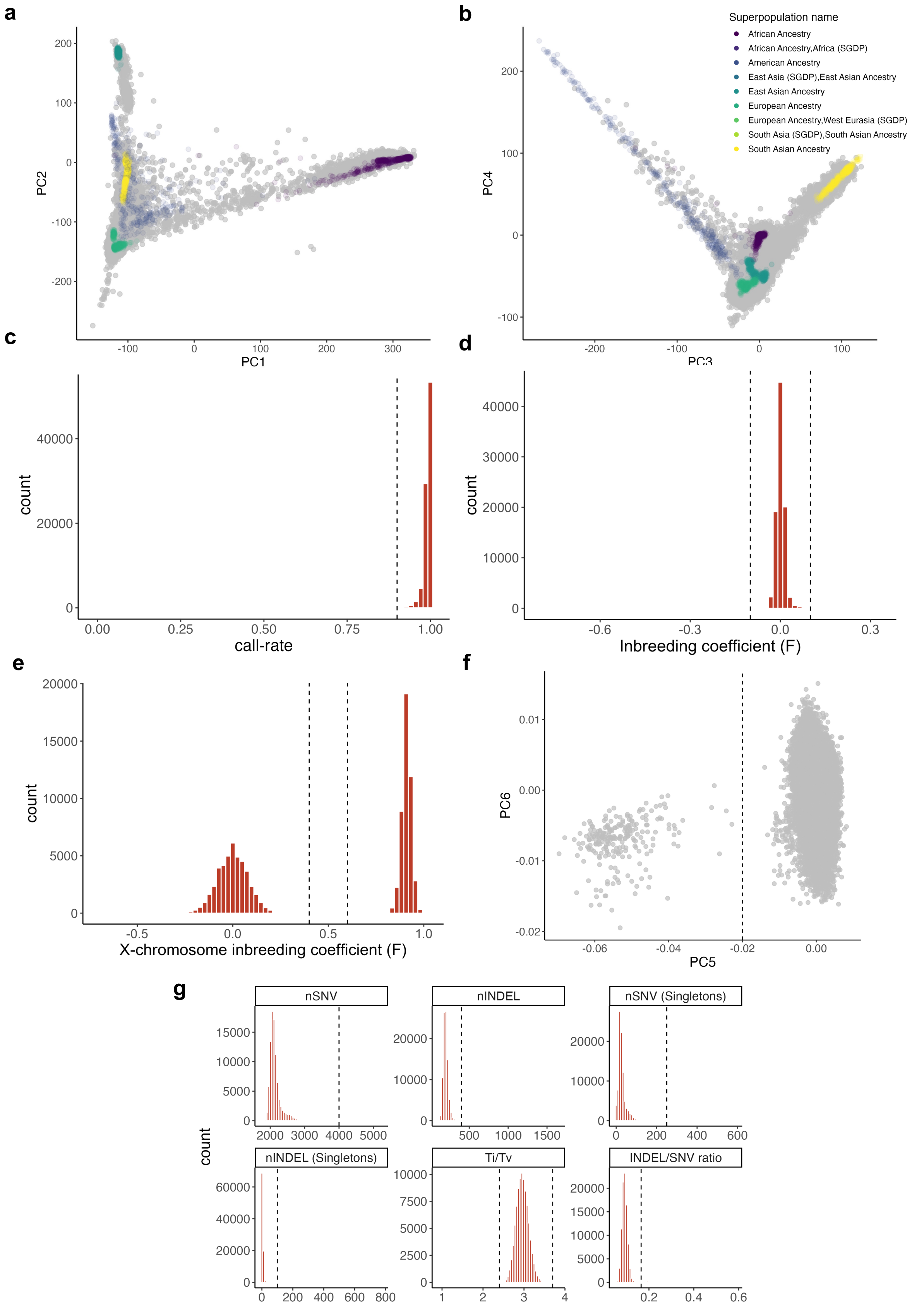

Supplementary Figure 2 | Sample quality control in the discovery cohort. a-b, Samples were projected onto the PCA coordinates of a reference ancestry space consisting of 1000

Genomes samples. The 94,545 samples included in this study are represented by grey dots, the colored dots indicate the 1000 Genomes samples (colored by superpopulation label) on which the study samples were projected. **c,** Distribution of sample call-rates, samples having a call rate < 0.9 were excluded. **d,** Inbreeding coefficient, samples with F-values <-0.1 or >0.1 were excluded. **e,** X-chromosome homozygosity (inbreeding coefficient), samples with ambiguous sex (0.4 < F < 0.6) or where genetically predicted sex did not match reported sex were excluded (F < 0.4 = female; F > 0.6 = male). **f,** Principal component analysis (PCA). A distinct cluster was identified on the fifth principal component, leading to the exclusion of samples with PC5 values less than -0.02. **g,** Total variant counts distributions. Samples were excluded if they exceeded one of the following thresholds: nSNV > 4000, nINDEL > 400, nSNV (Singletons) > 250, nINDEL (Singletons) > 100, Ti/Tv ratio < 2.4 or > 3.7, or INDEL/SNV ratio > 0.165.

a

Filters	MND cases	Controls
Initial cohort	15,862	78,683
Ancestry	14,857	75,587
Call-rate	14,439	75,466
Heterozygosity	14,349	75,386
Sex check	14,285	75,327
Total counts	14,195	73,879
Relatedness	13,140	70,007
PCA	13,138	69,775
Final cohort	13,138	69,775

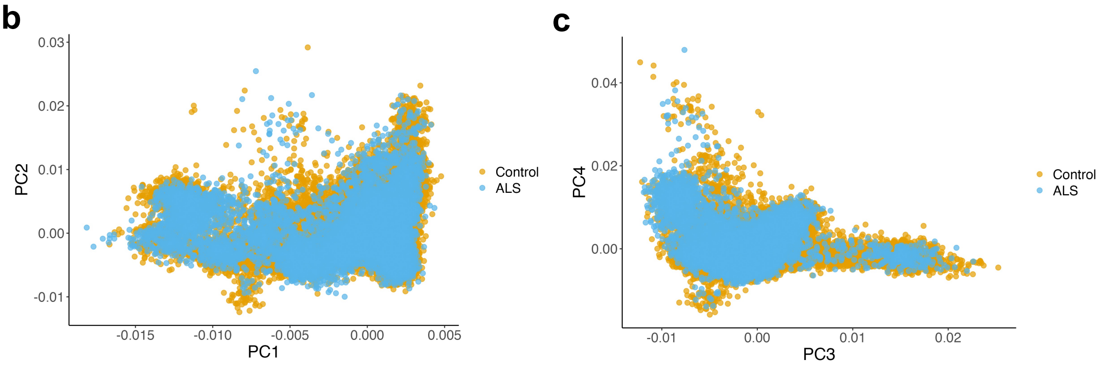

Supplementary Figure 3 | Discovery analysis cohort including 13,138 ALS cases and 69,775 controls. a, Successive sample quality control (QC) steps. First, individuals of broad European ancestry were retained. Subsequently, individuals were excluded if they exhibited low call rates (< 0.9), outlying heterozygosity rates (inbreeding $F < -0.1$ or $F > 0.1$), a genetic sex prediction inconsistent with reported sex, outlying counts of SNVs, INDELs, or singletons, as well as outlying values in Ti/Tv or SNV/INDEL ratios. Finally, individuals with ≤2nd degree relatedness (one member of each pair is kept) or with outlying values on the first five principal components were excluded. **b,** Principal component analysis of the final cohort consisting of 13,138 patients with ALS and 69,775 controls.

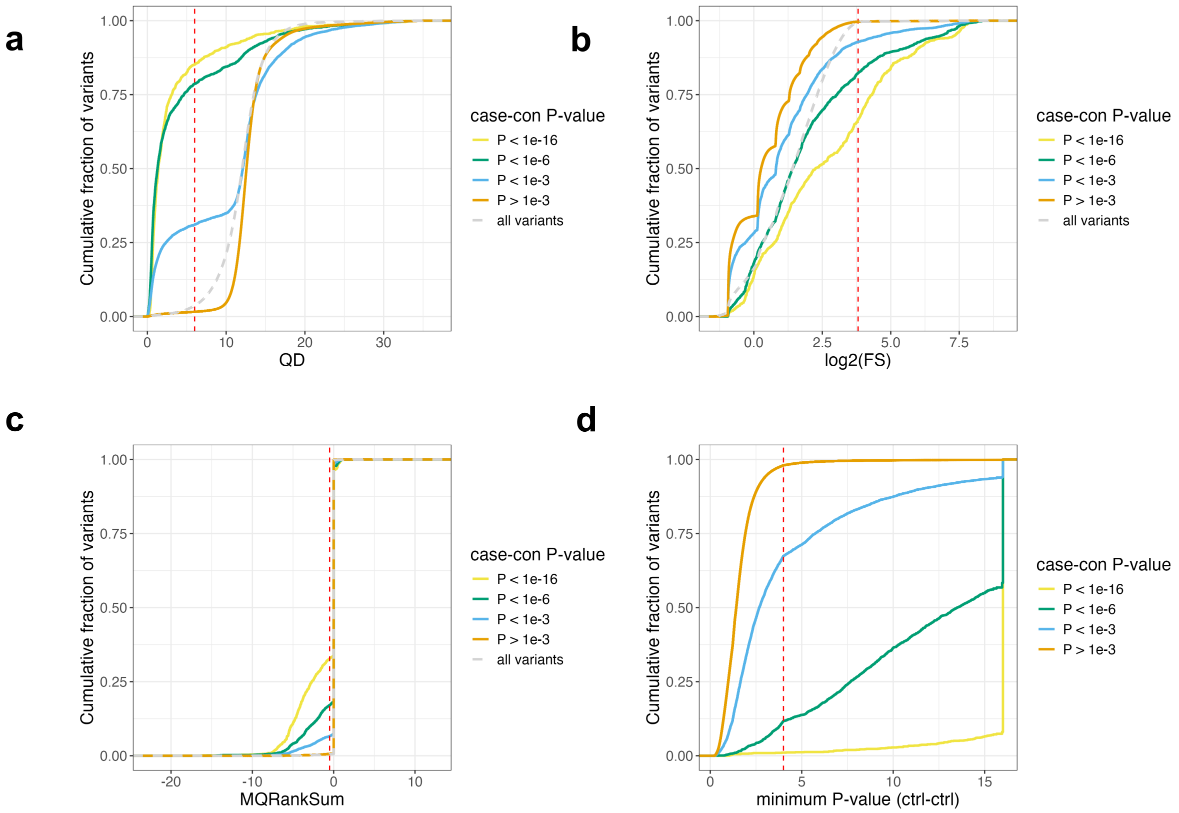

e

	SNV	INDEL	High-impact	Moderate- or high impact	Synonymous
All	20,644,578	4,438,363	652,624	5,190,152	2,100,579
Basic QC	5,536,528	521,013	261,664	2,665,539	1,142,873
Strict QC	4,944,044	263,094	214,488	2,367,861	1,039,192

Supplementary Figure 4 | Variant quality control in the discovery cohort. a-d, Calibration of quality score thresholds for variants passing basic quality control (VQSR-pass, per-supercohort call-rate > 0.9 and Hardy-Weinberg equilibrium *P*-value in controls > 0.0001). The figures show the cumulative fraction of variants, grouped by case-control *P*-value (two-tailed) estimated using Firth's logistic regression with profile penalized likelihood confidence intervals. Thresholds were chosen to retain the majority of variants while excluding a significant proportion of low-quality variants strongly associated with case-control status. Specifically, the following thresholds were set: QD ≥ 6, MQRankSum ≥ -0.5, FS ≤ 14, and ctrl-ctrl minimum *P*-value ≥ 0.0001. **e,** Overview of variant quality control steps. The first row shows the total number of called variants, the second row shows the number of variants passing basic QC, and the third row shows the number of variants that passed strict QC and were used in subsequent analyses.

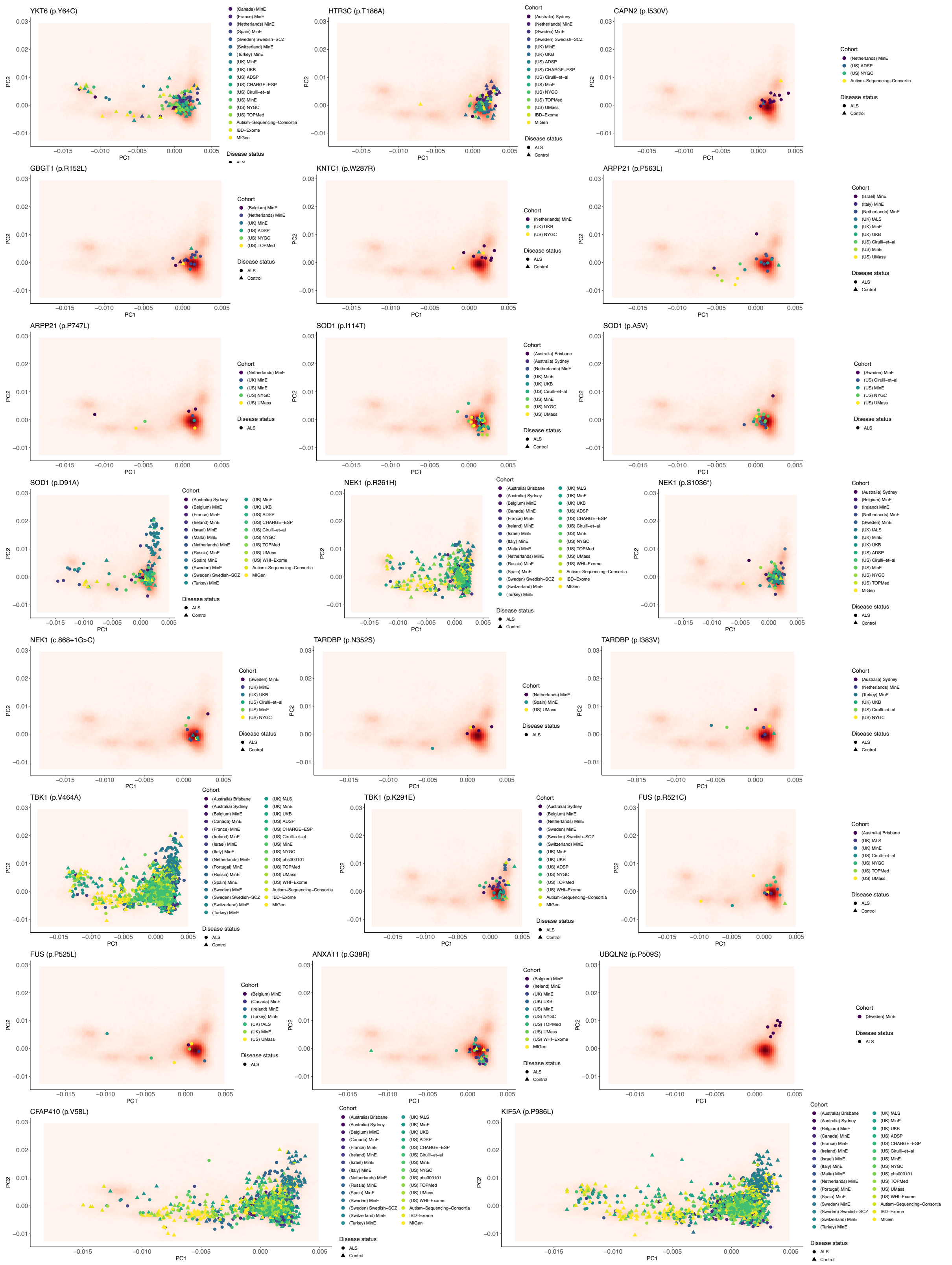

Supplementary Figure 5 | Single variant geographical carrier distributions. Density contours of the first two principal components are shown for the full study cohort. Overlaid on this background are the individuals carrying the respective variant, with points colored by their cohort of origin.

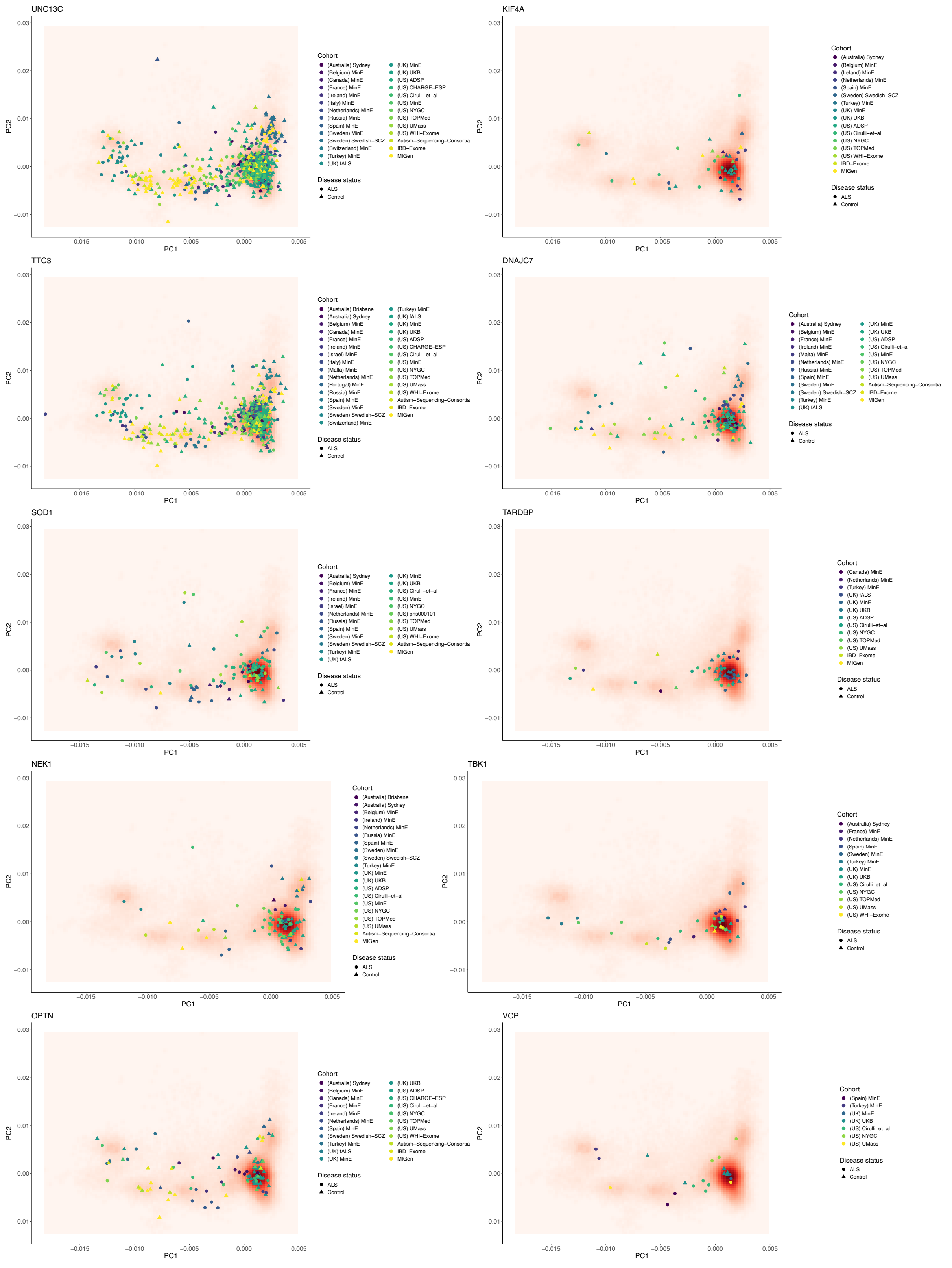

Supplementary Figure 6 | Ultra rare variant burden geographical carrier distributions. Density contours of the first two principal components are shown for the full study cohort. Overlaid on this background are the individuals carrying at least one ultra-rare variant in the respective gene, with points colored by their cohort of origin.

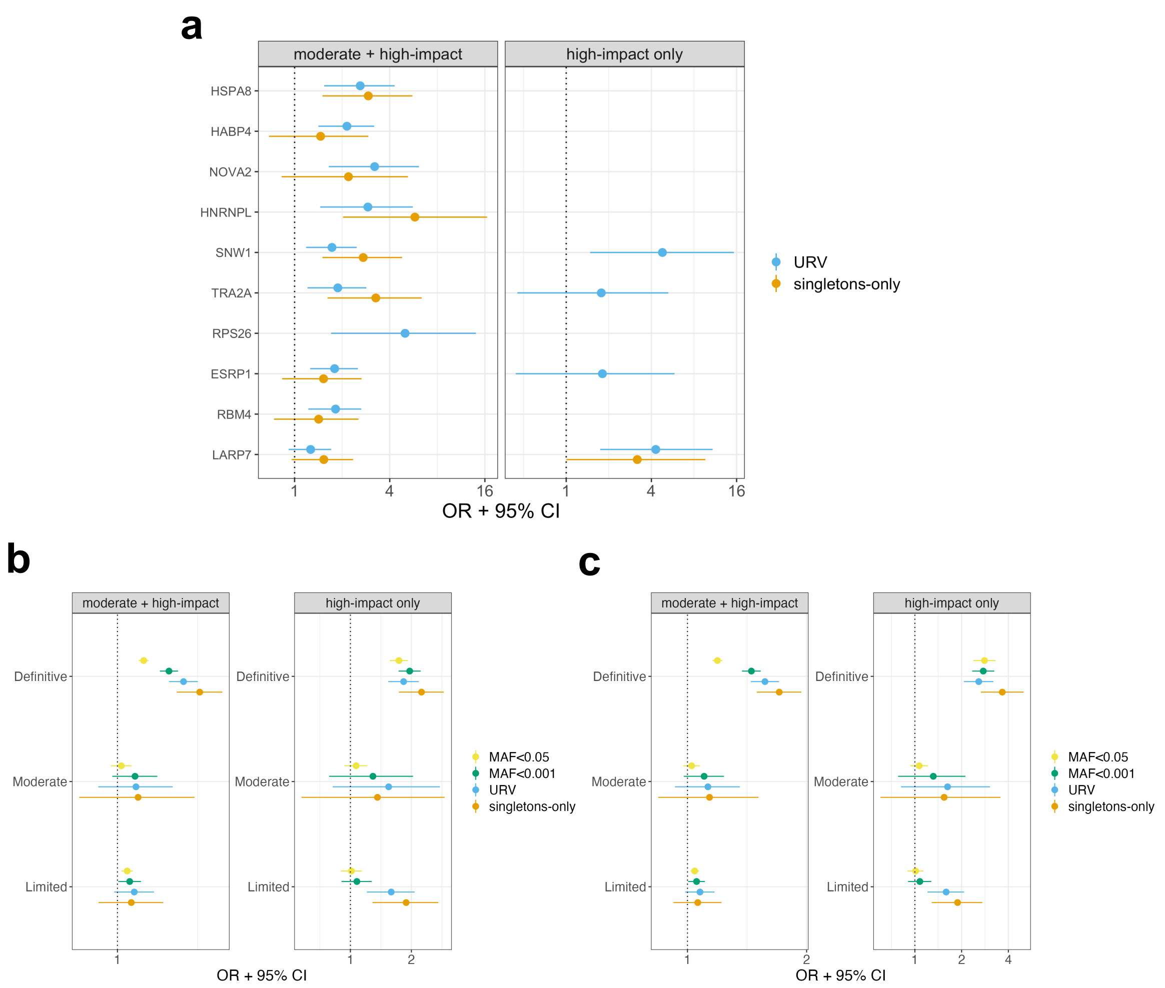

Supplementary Figure 7 | Ultra-rare gene set burden analyses. a, Forest plots depicting the odds ratio (OR) and 95% confidence intervals (y-axis) estimated using Firth's logistic regression of ultra-rare gene burden results for the ten most significant genes within the exome-wide significant gene sets ('GOBP: regulation of mRNA splicing via spliceosome' and 'GOBP: regulation of RNA splicing'). **b-c,** Forest plots depicting the odds ratio (OR) and 95% confidence intervals (y-axis) estimated using Firth's logistic regression for gene set burden of qualifying variants stratified by evidence level (x-axis). **b,** Analysis of all 51 genes curated for ALS spectrum disorders. **c,** Analysis restricted to 42 genes specifically associated with ALS. The 'Disputed' and 'Strong' categories were excluded from the analysis as they each contained only one gene.

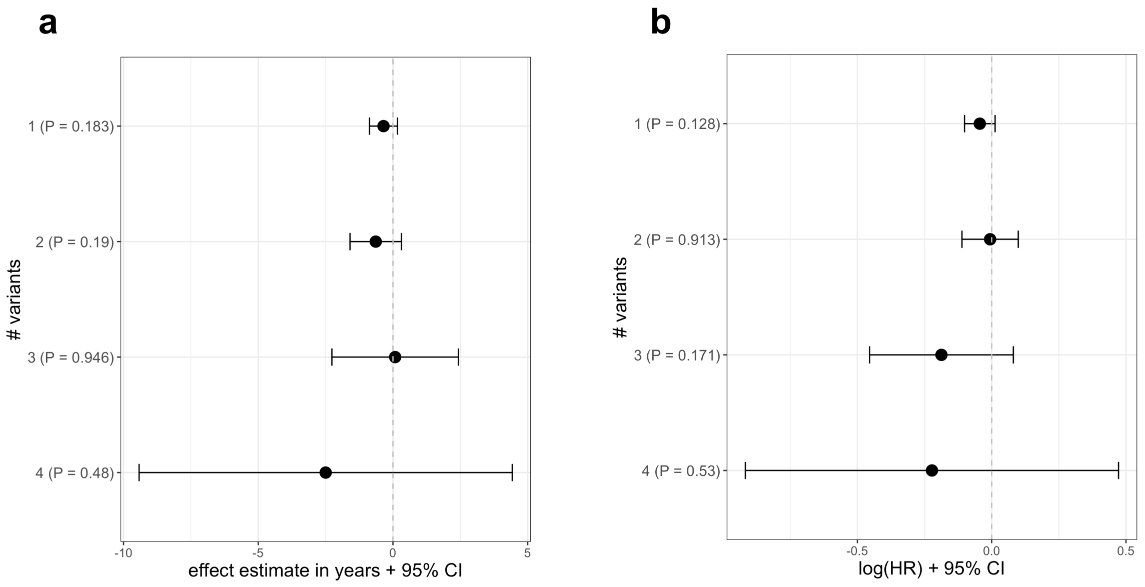

Supplementary Figure 8 | Cumulative burden analyses of age of onset and survival. Cumulative burden of carrying multiple high- or moderate-impact variants among Definitive ALS genes as curated by the GCEP. **a,** age of onset: shown are the effect estimates in years (center) and 95% confidence intervals (CI; error bars) (x-axis), stratified by the number of risk variants carried (y-axis). **b,** survival: shown are the log-transformed hazard ratios (center) and 95% confidence intervals (error bars) stratified by the number of risk variants carried (y-axis).

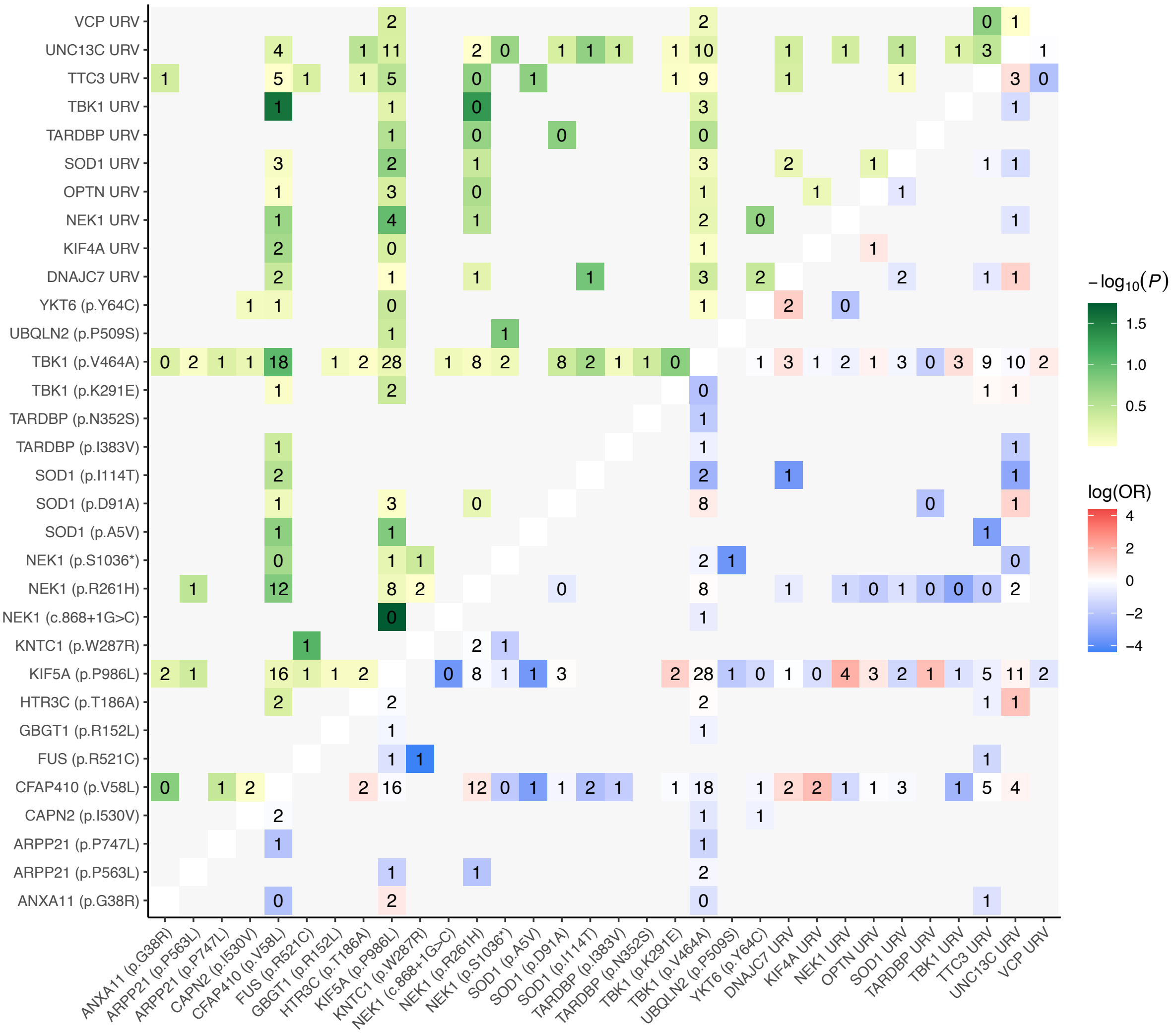

Supplementary Figure 9 | Variant co-occurrence heatmap and interaction analyses. Heatmap displaying the observed co-occurrences in ALS patients among variant pairs. For genes identified in the URV burden analysis, an individual is considered a carrier if they had at least one qualifying variant in that gene. Statistical interactions were estimated using Firth's logistic regression in the full case-control cohort. For each pair of variants, we fitted a model including their main effects and their interaction term, adjusting for the same covariates as used in the single variant analyses.The lower triangle shows the log-transformed odds-ratio, where red indicates a synergistic effect (positive interaction term) and blue indicates an antagonistic effect (negative interaction term). The upper triangle shows the statistical significance (-log$_{10}$(*P*-value)), with darker green indicating a more significant *P*-value. Pairs for which there was no co-occurrence in the full dataset (cases and controls) are greyed out. *P*-values are two-tailed and presented uncorrected for multiple testing.

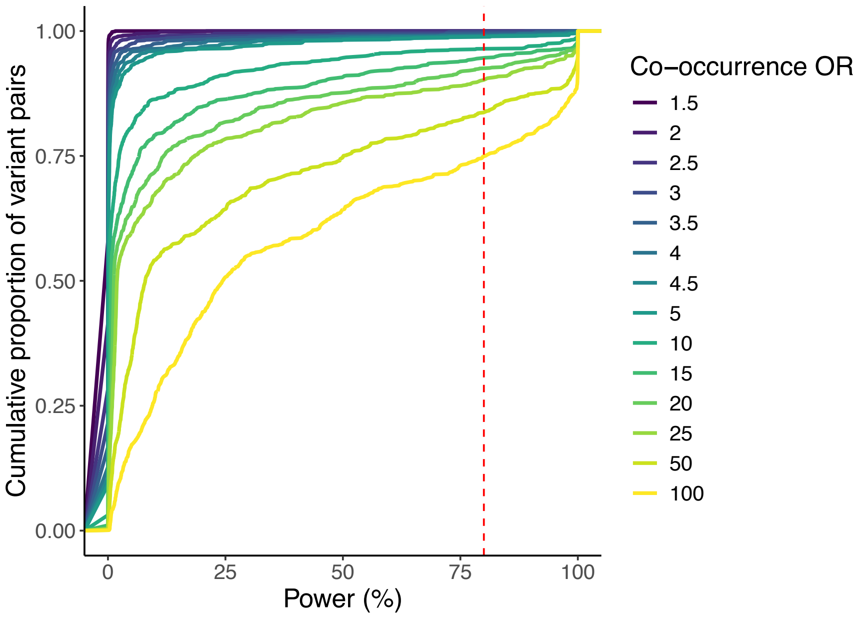

Supplementary Figure 10 | Variant cooccurrence power analyses. Co-occurrence power analyses among all tested pairs, with varying co-occurrence odds-ratios. Empirical cumulative distribution of statistical power (x-axis) to detect a significant excess or absence of co-occurrence between variant pairs. Each line represents the distribution of power across all tested pairs for a given co-occurrence odds ratio (OR). The y-axis shows the cumulative proportion of pairs with power at or below the corresponding power.

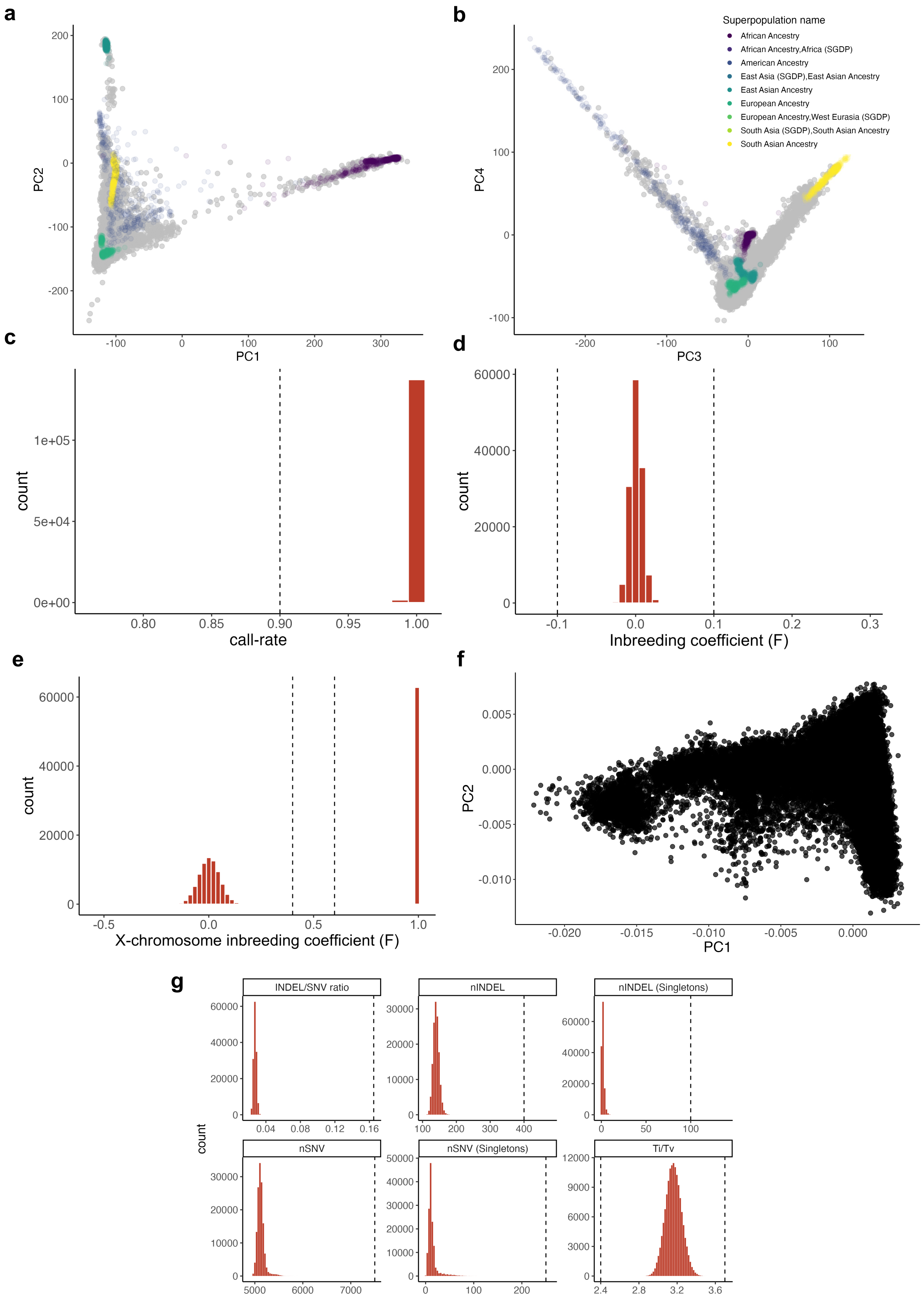

Supplementary Figure 11 | Sample quality control in the replication cohort. a-b, Samples were projected onto the PCA coordinates of a reference ancestry space consisting of

1000 Genomes samples. The 139,227 samples included in this cohort are represented by grey dots, the colored dots indicate the 1000 Genomes samples (colored by superpopulation label) on which the study samples were projected. **c,** Distribution of sample call-rates, samples having a call rate < 0.9 were excluded. **d,** Inbreeding coefficient, samples with *F*-values <-0.1 or >0.1 were excluded. **e,** X-chromosome homozygosity (inbreeding coefficient), samples with ambiguous sex (0.4 < *F* < 0.6) or where genetically predicted sex did not match reported sex were excluded (*F* < 0.4 = female; *F* > 0.6 = male). **f,** Principal component analysis (PCA). **g,** Total variant counts distributions. Samples were excluded if they exceeded one of the following thresholds: nSNV > 7500, nINDEL > 400, nSNV (Singletons) > 250, nINDEL (Singletons) > 100, Ti/Tv ratio < 2.4 or > 3.7, or INDEL/SNV ratio > 0.165.

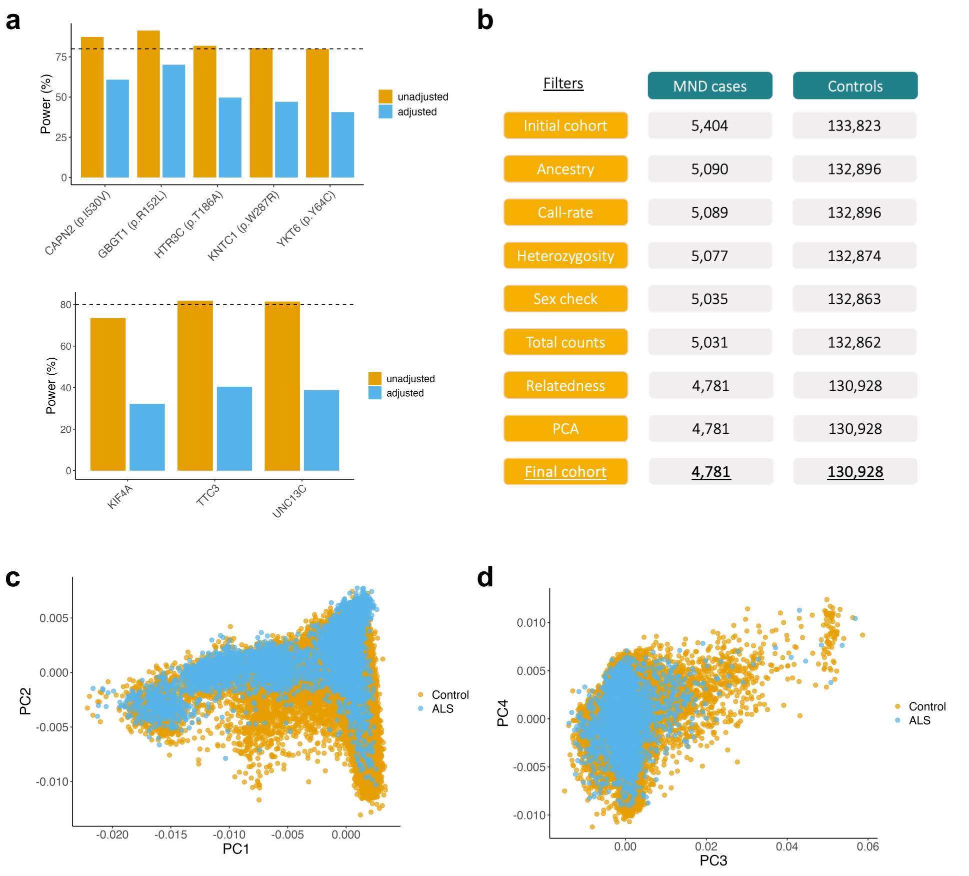

Supplementary Figure 12 | Power analyses and final replication analysis cohort including 4,781 patients with ALS and 130,928 controls.

a, Power analyses for the presented replication cohort including 4,781 patients with ALS and 130,928 controls. Bars represent statistical power, which was calculated through 10,000 simulations in which alleles were drawn from the binomial distribution, using Firth's logistic

regression to test for association with disease status. Of note, these power analyses assume no covariate adjustment, and actual power may be lower due to covariate inclusion. Orange bars indicate power analyses based on odds-ratios estimated from the discovery cohort, blue bars indicate power analyses where these odds-ratios were adjusted for winner's curse bias. The upper panel indicates the power analyses for the candidate single variants, and the lower panel indicates the power analyses for the candidate genes from the URV gene burden analyses.

b, Successive sample quality control (QC) steps. First, individuals of broad European ancestry were retained. Subsequently, individuals were excluded if they exhibited low call rates (< 0.9), outlying heterozygosity rates (inbreeding $F < -0.1$ or $F > 0.1$), a genetic sex prediction inconsistent with reported sex, outlying counts of SNVs, INDELs, or singletons, as well as outlying values in Ti/Tv or SNV/INDEL ratios. Finally, individuals with ≤2nd degree relatedness were excluded (one member of each pair is kept). Additionally, individuals that were duplicates or related up to the second degree to any sample in the discovery cohort were excluded. **c-d,** Principal component analysis of the final cohort consisting of 4,781 patients with ALS and 130,928 controls.

Consortium Authors

Project MinE ALS sequencing consortium

Philip Van Damme[65], Philippe Corcia[34], Philippe Couratier[36], Patrick Vourc'h[35], Orla Hardiman[57], Russell L. McLaughlin[12], Marc Gotkine[10], Vivian Drory[9], Nicola Ticozzi[54,55], Vincenzo Silani[54,55], Jan H. Veldink[1], Leonard H. Van den Berg[1], Mamede de Carvalho[40], Jesus S. Mora Pardina[32], Monica Povedano[33], Peter M. Andersen[44], Markus Weber[37], Nazli A. Başak[41], Ammar Al-Chalabi[3], Chris E. Shaw[3], Pamela J. Shaw[5,6], Karen E. Morrison[66], John E. Landers[64], Jonathan D. Glass[63], Clifton L. Dalgard[62]

NYGC ALS consortium

Hemali Phatnani[58,59,60], Justin Kwan[67,68], Dhruv Sareen[69], James R. Broach[70], Zachary Simmons[71], Ximena Arcila-Londono[72], Edward B. Lee[73], Vivianna M. Deerlin[73], Neil A. Shneider[74], Ernest Fraenkel[75], Lyle W. Ostrow[76], Frank Baas[77], Noah Zaitlen[78], James D. Berry[79], Andrea Malaspina[80], Pietro Fratta[81], Gregory A. Cox[82], Leslie M. Thompson[83], Steve Finkbeiner[84], Efthimios Dardiotis[85], Timothy M. Miller[86], Siddharthan Chandran[87], Suvankar Pal[87], Eran Hornstein[88], Daniel J. MacGowan[89], Terry Heiman-Patterson[90], Molly G. Hammell[91], Nikolaos. A. Patsopoulos[92], Oleg Butovsky[93], Joshua Dubnau[94], Avindra Nath[95], Robert Bowser[96], Matthew Harms[97], Eleonora Aronica[98], Mary Poss[99], Jennifer Phillips-Cremins[100], John Crary[101], Nazem Atassi[102], Dale J. Lange[103], Darius J. Adams[104], Leonidas Stefanis[105,106], Marc Gotkine[107], Robert H. Baloh[108,109], Suma Babu[110], Towfique Raj[111], Sabrina Paganoni[112], Ophir Shalem[113,114], Colin Smith[115,116], Bin Zhang[117], Thomas Blanchard[68], Brent Harris[118], Iris Broce[119], Vivian Drory[120], John Ravits[121], Corey McMillan[122], Vilas Menon[123], Lani Wu[124], Steven Altschuler[124], Yossef Lerner[125], Rita Sattler[126], Kendall Van Keuren-Jensen[127], Orit Rozenblatt-Rosen[128], Kerstin Lindblad-Toh[128], Katharine Nicholson[129], Peter Gregersen[130], Jeong-Ho Lee[131], Matt Brauer[132], Tara Nickerson[132], Shameek Biswas[133], Kimberly A Wilson[133], Sulev Koks[134], Stephen Muljo[135], Bryan J. Traynor[136], Robert Moccia[137], Seng Cheng[137], Andrew Deubler[138], Giovanni Coppola[138], Mickey Atwal[138], Michael Cantor[138], William Salerno[138], Eli Stahl[138], Matt Anderson[138], David Frendewey[138], Daphne Koller[139], Mary Rozenman[139]

67: Department of Neurology, Lewis Katz School of Medicine, Temple University, Philadelphia, PA
68: University of Maryland Brain and Tissue Bank and NIH NeuroBioBank
69: Cedars-Sinai Department of Biomedical Sciences, Board of Governors Regenerative Medicine Institute and Brain Program, Cedars-Sinai Medical Center, and Department of Medicine, University of California, Los Angeles, CA
70: Department of Biochemistry and Molecular Biology, Penn State Institute for Personalized Medicine, The Pennsylvania State University, Hershey, PA

71: Department of Neurology, The Pennsylvania State University, Hershey, PA
72: Department of Neurology, Henry Ford Health System, Detroit, MI
73: Department of Pathology and Laboratory Medicine, Perelman School of Medicine, University of Pennsylvania, Philadelphia, PA
74: Department of Neurology, Center for Motor Neuron Biology and Disease, Institute for Genomic Medicine, Columbia University, New York, NY
75: Department of Biological Engineering, Massachusetts Institute of Technology, Cambridge, MA
76: Department of Neurology, Johns Hopkins School of Medicine, Baltimore, MD
77: Department of Neurogenetics, Academic Medical Centre, Amsterdam and Leiden University Medical Center, Leiden, The Netherlands
78: Department of Medicine, Lung Biology Center, University of California, San Francisco, CA
79: ALS Multidisciplinary Clinic, Neuromuscular Division, Department of Neurology, Harvard Medical School, and Neurological Clinical Research Institute, Massachusetts General Hospital, Boston, MA
80: Centre for Neuroscience and Trauma, Blizard Institute, Barts and The London School of Medicine and Dentistry, Queen Mary University of London, London, and Department of Neurology, Basildon University Hospital, Basildon, United Kingdom
81: Institute of Neurology, National Hospital for Neurology and Neurosurgery, University College London, London, United Kingdom
82: The Jackson Laboratory, Bar Harbor, ME
83: Department of Psychiatry & Human Behavior, Department of Biological Chemistry, School of Medicine, and Department of Neurobiology and Behavior, School of Biological Sciences, University California, Irvine, CA
84: Taube/Koret Center for Neurodegenerative Disease Research, Roddenberry Center for Stem Cell Biology and Medicine, Gladstone Institute
85: Department of Neurology & Sensory Organs, University of Thessaly, Thessaly, Greece
86: Department of Neurology ,Washington University in St. Louis, St. Louis, MO
87: Centre for Clinical Brain Sciences, Anne Rowling Regenerative Neurology Clinic, Euan MacDonald Centre for Motor Neurone Disease Research, University of Edinburgh, Edinburgh, United Kingdom
88: Department of Molecular Genetics, Weizmann Institute of Science, Rehovot, Israel
89: Department of Neurology, Icahn School of Medicine at Mount Sinai, New York, NY
90: Center for Neurodegenerative Disorders, Department of Neurology, the Lewis Katz School of Medicine, Temple University, Philadelphia, PA
91: Cold Spring Harbor Laboratory, Cold Spring Harbor, NY
92: Computer Science and Systems Biology Program, Ann Romney Center for Neurological Diseases, Department of Neurology and Division of Genetics in Department of Medicine, Brigham and Women’s Hospital, Boston, MA, Harvard Medical School, Boston, MA, and Program in Medical and Population Genetics, Broad Institute, Cambridge, MA
93: Ann Romney Center for Neurologic Diseases, Brigham and Women's Hospital, Harvard Medical School, Boston, MA
94: Department of Anesthesiology, Stony Brook University, Stony Brook, NY
95: Section of Infections of the Nervous System, National Institute of Neurological Disorders and Stroke, NIH, Bethesda, MD

96: Department of Neurology, Barrow Neurological Institute, St. Joseph's Hospital and Medical Center, Department of Neurobiology, Barrow Neurological Institute, St. Joseph's Hospital and Medical Center, Phoenix, AZ
97: Department of Neurology, Division of Neuromuscular Medicine, Columbia University, New York, NY
98: Department of Neuropathology, Academic Medical Center, University of Amsterdam, Amsterdam, The Netherlands
99: Department of Biology and Veterinary and Biomedical Sciences, The Pennsylvania State University, University Park, PA
100: New York Stem Cell Foundation, Department of Bioengineering, School of Engineering and Applied Sciences, University of Pennsylvania, Philadelphia, PA
101: Department of Pathology, Fishberg Department of Neuroscience, Friedman Brain Institute, Ronald M. Loeb Center for Alzheimer's Disease, Icahn School of Medicine at Mount Sinai, New York, NY
102: Department of Neurology, Harvard Medical School, Neurological Clinical Research Institute, Massachusetts General Hospital, Boston, MA
103: Department of Neurology, Hospital for Special Surgery and Weill Cornell Medical Center, New York, NY
104: Medical Genetics, Atlantic Health System, Morristown Medical Center, Morristown, NJ, and Overlook Medical Center, Summit, NJ
105: Center of Clinical Research, Experimental Surgery and Translational Research, Biomedical Research Foundation of the Academy of Athens (BRFAA), 4 Soranou Efesiou Street, 11527, Athens, Greece
106: 1st Department of Neurology, Eginition Hospital, Medical School, National and Kapodistrian University of Athens, Athens, Greece
107: Neuromuscular/EMG service and ALS/Motor Neuron Disease Clinic , Hebrew University-Hadassah Medical Center, Jerusalem, Israel
108: Board of Governors Regenerative Medicine Institute, Los Angeles, CA
109: Department of Neurology, Cedars-Sinai Medical Center, Los Angeles, CA
110: Neurological Clinical Research Institute, Massachusetts General Hospital, Boston, MA
111: Departments of Neuroscience, and Genetics and Genomic Sciences, Ronald M. Loeb Center for Alzheimer's disease, Icahn School of Medicine at Mount Sinai, New York, NY
112: Harvard Medical School, Department of Physical Medicine & Rehabilitation, Spaulding Rehabilitation Hospital, Boston, MA
113: Center for Cellular and Molecular Therapeutics, Children's Hospital of Philadelphia, Philadelphia, PA
114: Department of Genetics, Perelman School of Medicine, University of Pennsylvania, Philadelphia, PA
115: Centre for Clinical Brain Sciences, University of Edinburgh, Edinburgh, UK
116: Euan MacDonald Centre for Motor Neurone Disease Research, University of Edinburgh, Edinburgh, UK
117: Department of Genetics and Genomic Sciences, Icahn Institute of Data Science and Genomic Technology, Icahn School of Medicine at Mount Sinai, New York, NY
118: Department of Neuropathology, Georgetown Brain Bank, Georgetown Lombardi

Comprehensive Cancer Center, Georgetown University Medical Center, Washington DC
119: Neuroradiology Section, Department of Radiology and Biomedical Imaging, University of California, San Francisco, San Francisco, CA
120: Neuromuscular Diseases Unit, Department of Neurology, Tel Aviv Sourasky Medical Center, Sackler Faculty of Medicine, Tel-Aviv University, Tel-Aviv, Israel
121: Department of Neuroscience, University of California San Diego, La Jolla, CA
122: Department of Neurology, University of Pennsylvania Perelman School of Medicine, Philadelphia, PA
123: Department of Neurology, Columbia University Medical Center, New York, NY
124: Department of Pharmaceutical Chemistry, University of California San Francisco, San Francisco, CA
125: Hadassah Hebrew University
126: Department of Translational Neuroscience, Barrow Neurological Institute, Phoenix, Arizona
127: The Translational Genomics Research Institute (TGen), Phoenix, Arizona
128: Broad Institute, Cambridge, Massachusetts
129: Massachusetts General Hospital, Boston, Massachusetts
130: Institute of Molecular Medicine, Feinstein Institutes for Medical Research, Northwell Health, Manhasset, New York
131: Korea Advanced Institute of Science and Technology (KAIST), Daejeon, South Korea
132: Maze Therapeutics
133: Bristol-Myers Squibb
134: Perron Institute for Neurological and Translational Science
135: Integrative Immunobiology Section, National Institute of Allergy and Infectious Disease, NIH
136: Neuromuscular Disease Research Section, National Institute of Aging
137: Pfizer
138: Regeneron
139: Insitro

FALS Sequencing Consortium

Peter C. Sapp[140], Claire S. Leblond[141], Diane McKenna-Yasek[142], Kevin P. Kenna[2], Bradley N. Smith[3], Simon Topp[4], Jack Miller[143], Athina Gkazi[143], Ammar Al-Chalabi[3], Leonard H. Van den Berg[1], Jan H. Veldink[1], Vincenzo Silani[54,55], Nicola Ticozzi[54,55], John E. Landers[64], Frank Baas[144], Chris E. Shaw[3], Jonathan D. Glass[63], Guy A. Rouleau[51], Robert Brown[140], Matthew B. Harms[61,58,59]

140: Department of Neurology, University of Massachusetts Medical School, Worcester, MA, USA.
141: Human Genetics and Cognitive Functions, Institut Pasteur, CNRS UMR3571, Institut Universitaire de France, Université Paris Cité, Paris, France.
142: Department of Neurology, University of Massachusetts Medical School, Worcester, MA 02125, USA.
143: Department of Clinical Neuroscience, King's College London Centre for Neurodegeneration

Research, Institute of Psychiatry, London SE5 8AF, UK.
144: Department of Clinical Genetics Leiden University Medical Center Leiden The Netherlands.

GTAC Consortium

Matthew B. Harms[61,58,59], Stanley Appel[145], Robert Baloh[146], Richard Bedlack[147], Siddharthan Chandran[148], Laura Foster[149], Stephen Goutman[150], Ericka Greene[145], Chafic Karam[151], David Lacomis[152], George Manousakis[153], Timothy Miller[154], Suvankar Pals[148], Dhruv Sareen[155], Alex Sherman[156], Zachary Simmons[157], Leo Wang[158]

145: Department of Neurology, Houston Methodist, Houston, Texas, United States of America.
146: Department of Neurology, Cedars Sinai, Los Angeles, California, United States of America.
147: Department of Neurology, Duke University, Durham, North Carolina, United States of America.
148: Department of Neurology, University of Edinburgh, Edinburgh, Scotland, UK.
149: Department of Neurology, University of Colorado, Aurora, Colorado, United States of America.
150: Department of Neurology, University of Michigan, Ann Arbor, Michigan, United States of America.
151: Department of Neurology, Oregon Health Sciences University, Portland, Oregon, United States of America.
152: Department of Neurology, University of Pittsburg Medical Center, Pittsburgh, Pennsylvania, United States of America.
153: Department of Neurology, University of Minnesota, Minneapolis, Minnesota, United States of America.
154: Department of Neurology, Washington University in St. Louis, St. Louis, Missouri, United States of America.
155: Cedars Sinai, Los Angeles, California, United States of America.
156: Massachusetts General Hospital, Boston, Massachusetts, United States of America.
157: Department of Neurology, The Pennsylvania State University, College of Medicine, Hershey, Pennsylvania, United States of America.
158: Department of Neurology, University of Washington, Seattle, Washington, United States of America.
