# Supplementary material for: Large-scale exome analyses reveal new rare variant contributions in amyotrophic lateral sclerosis
Source: Nat Genet. 2026 Mar 31;58(4):717–25. doi: 10.1038/s41588-026-02535-9 (PMC13083253; doi:10.1038/s41588-026-02535-9)
Supplement: Supplementary file 2 — Reporting Summary [file 41588_2026_2535_MOESM2_ESM.pdf]

nature portfolio

Corresponding author(s): J.H. Veldink, K.P. Kenna

Last updated by author(s): Nov 17, 2025

Reporting Summary

Nature Portfolio wishes to improve the reproducibility of the work that we publish. This form provides structure for consistency and transparency in reporting. For further information on Nature Portfolio policies, see our Editorial Policies and the Editorial Policy Checklist.

Statistics

For all statistical analyses, confirm that the following items are present in the figure legend, table legend, main text, or Methods section.

n/a	Confirmed	
☐	☒	The exact sample size (*n*) for each experimental group/condition, given as a discrete number and unit of measurement
☒	☐	A statement on whether measurements were taken from distinct samples or whether the same sample was measured repeatedly
☐	☒	The statistical test(s) used AND whether they are one- or two-sided<br>*Only common tests should be described solely by name; describe more complex techniques in the Methods section.*
☐	☒	A description of all covariates tested
☐	☒	A description of any assumptions or corrections, such as tests of normality and adjustment for multiple comparisons
☐	☒	A full description of the statistical parameters including central tendency (e.g. means) or other basic estimates (e.g. regression coefficient) AND variation (e.g. standard deviation) or associated estimates of uncertainty (e.g. confidence intervals)
☐	☒	For null hypothesis testing, the test statistic (e.g. *F*, *t*, *r*) with confidence intervals, effect sizes, degrees of freedom and *P* value noted<br>*Give P values as exact values whenever suitable.*
☒	☐	For Bayesian analysis, information on the choice of priors and Markov chain Monte Carlo settings
☒	☐	For hierarchical and complex designs, identification of the appropriate level for tests and full reporting of outcomes
☐	☒	Estimates of effect sizes (e.g. Cohen's *d*, Pearson's *r*), indicating how they were calculated

Our web collection on statistics for biologists contains articles on many of the points above.

Software and code

Policy information about availability of computer code

Data collection	All raw sequencing data was aligned to the GRCh38 reference genome using BWA-mem (v.2.2.1) according to the pipeline described by Regier et al. (implementation can be found at GitHub (https://github.com/maarten-k/realignment) and Zenodo (https://doi.org/10.5281/zenodo.10963076). Joint genotyping was performed using a uniform pipeline according to the GATK best practices (v. 4.2.6.1).
Data analysis	All raw sequencing data was aligned to the GRCh38 reference genome using BWA-mem (v.2.2.1) according to the pipeline described by Regier et al. (implementation can be found at GitHub (https://github.com/maarten-k/realignment) and Zenodo (https://doi.org/10.5281/zenodo.10963076). Joint genotyping was performed using a uniform pipeline according to the GATK best practices (v. 4.2.6.1). Handling and filtering of VCF files was performed using VCFtools (v. 0.1.16), BCFtools (v. 1.9) and PLINK (v. 1.90b6.21). Ancestry was estimated using LASER (v.2.04). Variants were annotated using Ensembl (GRCh38.105), snpEff (v.5.1d) and dbNSFP (v. 4.3a). Sample and variant quality control was performed using PLINK (v. 1.90b6.21) and RVAT (v. 0.2.0), while sample relatedness was inferred using KING (v. 2.2.7). Meta-analyses were performed using METAL (v. 2011-03-25). Gene ontology terms were summarized using the rrvgo R package (v. 1.18.0). All downstream analyses were performed using custom R code (performed in R 3.6.3) that we made available in the RVAT R package (v. 0.2.0) (available on GitHub: https://github.com/kennalab/rvat and Zenodo: https://doi.org/10.5281/zenodo.10973472). Other R packages used either as dependencies of RVAT or in other analyses and visualizations are ggplot2 (v. 3.4.2), ggrepel (v. 0.9.1), dplyr (v. 1.0.7), readr (v. 2.1.1), stringr (v. 1.4.0), tidyr (v. 1.1.4), magrittr (v. 2.0.1), kinship2 (v. 1.9.6), logistf (v. 1.25.0), SKAT (v. 2.2.5), SummarizedExperiment (v. 1.16.1), S4Vectors (v. 0.24.4), GenomicRanges (v. 1.38.0), IRanges (v. 2.20.2), libraDBI (v. 1.1.3), RSQLite (v. 2.3.1), survival (v. 3.1.8), winnerscurse (v. 0.1.1). Figures were generated using R 4.2.3, using rvat (v. 0.3.4), dplyr (v.1.1.4), ggplot (v. 3.5.1), readr (v. 2.15), ggrepel (v. 0.9.5), colorblindr (v. 0.1.0), stringr (v. 1.5.1), tidyr (v. 1.3.1), and magrittr (v. 2.0.3).

For manuscripts utilizing custom algorithms or software that are central to the research but not yet described in published literature, software must be made available to editors and reviewers. We strongly encourage code deposition in a community repository (e.g. GitHub). See the Nature Portfolio guidelines for submitting code & software for further information.

Data

Policy information about availability of data

All manuscripts must include a data availability statement. This statement should provide the following information, where applicable:

- Accession codes, unique identifiers, or web links for publicly available datasets
- A description of any restrictions on data availability
- For clinical datasets or third party data, please ensure that the statement adheres to our policy

Project MinE data are available here: https://www.projectmine.com/research/data-sharing/. dbGAP datasets used are available under the following accession numbers: ALS compute (phs003184); Alzheimer's Disease Sequencing Project (ADSP) (phs000572); Autism Sequencing Consortium (ASC) (phs000298); Sweden-Schizophrenia Population-Based Case-Control Exome Sequencing (phs000473); Inflammatory Bowel Disease Exome Sequencing Study (phs001076); Myocardial Infarction Genetics Exome Sequencing Consortium: Ottawa Heart Study (phs000806); Myocardial Infarction Genetics Exome Sequencing Consortium: Malmo Diet and Cancer Study (phs001101); Myocardial Infarction Genetics Exome Sequencing Consortium: U. of Leicester (phs001000); Myocardial Infarction Genetics Exome Sequencing Consortium: Italian Atherosclerosis Thrombosis and Vascular Biology (phs000814); NHLBI GO-ESP: Women's Health Initiative Exome Sequencing Project (WHI) - WHISP (phs000281); Building on GWAS for NHLBI diseases: The US CHARGE Consortium (CHARGE-S): CHS (phs000667); Building on GWAS for NHLBI Diseases: the US CHARGE Consortium (CHARGE-S): ARIC (phs000668); Building on GWAS for NHLBI diseases: the US CHARGE consortium (CHARGE-S): FHS (phs000651); NHLBI GO-ESP Family Studies: Idiopathic Bronchiectasis (phs000518); NHLBI GO-ESP: Family Studies (Hematological Cancers) (phs000632); NHLBI GO-ESP: Family Studies: (familial atrial fibrillation) (phs000362); NHLBI GO-ESP: Heart Cohorts Exome Sequencing Project (ARIC) (phs000398); NHLBI GO-ESP: Heart Cohorts Exome Sequencing Project (CHS) (phs000400); NHLBI GO-ESP: Heart Cohorts Exome Sequencing Project (FHS) (phs000401); NHLBI GO-ESP: Lung Cohorts Exome Sequencing Project (asthma) (phs000422); NHLBI GO-ESP: Lung Cohorts Exome Sequencing Project (COPDGene) (phs000296); GO-ESP: Family Studies (Thoracic aortic aneurysms leading to acute aortic dissections) (phs000347). NHLBI TOPMed: Genomic Activities such as Whole Genome Sequencing and Related Phenotypes in the Framingham Heart Study (phs000974); NHLBI TOPMed: Genetics of Cardiometabolic Health in the Amish (phs000956); NHLBI TOPMed: Genetic Epidemiology of COPD (COPDGene) (phs000951); NHLBI TOPMed: The Vanderbilt Atrial Fibrillation Registry (VU_AF) (phs001032); NHLBI TOPMed: Cleveland Clinic Atrial Fibrillation (CCAF) Study (phs001189); NHLBI TOPMed: Partners HealthCare Biobank (phs001024); NHLBI TOPMed - NHGRI CCDG: Massachusetts General Hospital (MGH) Atrial Fibrillation Study (phs001062); NHLBI TOPMed: Novel Risk Factors for the Development of Atrial Fibrillation in Women (phs001040); NHLBI TOPMed - NHGRI CCDG: The Vanderbilt AF Ablation Registry (phs000997); NHLBI TOPMed: Heart and Vascular Health Study (HVH) (phs000993); NHLBI TOPMed - NHGRI CCDG: Atherosclerosis Risk in Communities (ARIC) (phs001211); NHLBI TOPMed: The Genetics and Epidemiology of Asthma in Barbados (phs001143); NHLBI TOPMed: Women's Health Initiative (WHI) (phs001237); NHLBI TOPMed: Whole Genome Sequencing of Venous Thromboembolism (WGS of VTE) (phs001402); NHLBI TOPMed: Trans-Omics for Precision Medicine (TOPMed) Whole Genome Sequencing Project: Cardiovascular Health Study (phs001368). All participants gave written informed consent, and all studies were approved by the institutional review boards of the respective participating centers.

Research involving human participants, their data, or biological material

Policy information about studies with human participants or human data. See also policy information about sex, gender (identity/presentation), and sexual orientation and race, ethnicity and racism.

Reporting on sex and gender	Sex was included as a covariate in all analyses that included individual-level data (single variant analyses; gene, domain, and gene set-based ultra-rare burden analyses). Self-reported sex was used if available, otherwise genetically inferred sex (plink software) was used.
Reporting on race, ethnicity, or other socially relevant groupings	No socially constructed or socially relevant categorization variables, such as self-reported race or ethnicity, were used for participant categorization or analysis in this manuscript. Genetic ancestry was inferred for all participants using the LASER software (Supplementary Figs. 2 and 11), and individuals of predominantly European ancestry were retained to ensure coverage of both cases and controls across ancestry space. The first ten principal components derived from common variants (MAF > 0.01) were included as covariates in all presented analyses that included individual-level data (single variant analyses; gene, domain, and gene set-based ultra-rare burden analyses).
Population characteristics	The discovery cohort (post-QC) included 13,138 patients with ALS (7,836 male, 5,302 female) and 69,775 control subjects (34,778 male and, 34,997 female)<br>The replication cohort (post-QC) included 4,781 patients with ALS (2,833 male, 1,948 female) and 130,928 control subjects (58,516 male and 72,412 female).
Recruitment	The discovery cohort included 15,862 patients with ALS and 78,683 control subjects, totaling 94,545 subjects of which 21,102 were subjected to whole-genome sequencing (WGS) and 73,443 to whole-exome sequencing (WXS). Case cohorts included the Project MinE ALS sequencing consortium (7,614 cases, 2,605 controls), the NYGC ALS Consortium (2,650 cases, 342 controls), the ALS Sequencing Consortium (2,851 cases), two cohorts from the FALS consortium (1,277 cases; phs001585), the NIH Exome Sequencing of FALS Project (194 cases; phs000101) two Australian cohorts described in (Garton et al., 2017) (125 cases and 18 controls) and (McCann et al., 2021) (568 cases), and a Chinese MND cohort described in (Gratten et al., 2017) (583 cases, 182 controls). All patients were diagnosed with definite, probable, or probable laboratory-supported ALS according to the revised El Escorial Criteria. Control cohorts included 7,323 samples from the NHLBI TOPMed research programme, 49,981 samples from the UK Biobank, and 18,232 samples across 7 cohorts from dbGAP.<br><br>The replication cohort included 5,404 patients with ALS and 133,823 control subjects, totaling 139,227 subjects, of which all were subjected to WGS. Cohorts include the Project MinE ALS sequencing consortium (1,510 cases, 169 controls), the NYGC ALS consortium (1,257 cases, 69 controls), ALS compute (1,870 cases, 1,820 controls; phs003184) and the UK Biobank (767 cases, 131,765 controls). During sample quality control, individuals who were duplicates or related up to the second degree to any participant in the discovery cohort were excluded.
Ethics oversight	This study was approved by the institutional review boards of all participating centers, written informed consent for research

Ethics oversight	was obtained from each individual, and the study was approved by the Medical Ethical Testing Committee NedMec and the Biobanks Testing Committee of UMC Utrecht.

Note that full information on the approval of the study protocol must also be provided in the manuscript.

Field-specific reporting

Please select the one below that is the best fit for your research. If you are not sure, read the appropriate sections before making your selection.

☒ Life sciences ☐ Behavioural & social sciences ☐ Ecological, evolutionary & environmental sciences

For a reference copy of the document with all sections, see nature.com/documents/nr-reporting-summary-flat.pdf

Life sciences study design

All studies must disclose on these points even when the disclosure is negative.

Sample size	The discovery dataset included 15,862 patients with ALS and 78,683 control subjects. After quality control and relatedness filtering the discovery analysis cohort included 13,138 patients with ALS and 69,775 controls. The sample size was not predetermined; instead, we assembled the largest possible sample size to optimize power. In the manuscript we show that we were adequately powered to detect most of the known ALS genes, including ultra-rare variants that have hitherto not been detected in case-control studies.<br>The replication cohort included 5,404 patients with ALS and 133,823 control subjects. After quality control and relatedness filtering the replication analysis cohort included 4,781 patients with ALS and 130,928 controls. Power analyses showed that this sample size provides between 32¬--91% statistical power for replication across candidate variants and genes.
Data exclusions	Samples and variants were subjected to stringent quality control as detailed in the Methods section. Moreover, we retained only unrelated individuals (>= 2nd degree), and variants were included when predicted to have a moderate- or high-impact as predicted by the snpEff software and were either low-frequency (MAF < 0.05) in the single variant analyses or ultra-rare (<=5 carriers) in the burden analyses.
Replication	For replication, we generated a cohort comprising 4,781 patients with ALS and 130,928 controls after applying stringent quality control criteria identical to those used in the discovery set. This confirmed the single variant association in YKT6 (p.Y64C) at replication-wide significance. In addition, the single variant associations in HTR3C (p.T186A) KNTC1 (p.W287R), and GBGT1(p.R152L) meta-analysis of discovery and replication cohorts showed greater significance than the discovery phase alone. TTC3, KIF4A, UNC13C and CAPN2 could not be replicated with available samples and are thus explicitly presented as candidates for future research that only achieve significance in the discovery phase.
Randomization	This study used a case-control design, and therefore randomization is not applicable. All analyses that included individual-level data were adjusted for sex, ten principal components and the total number of synonymous variants in each individual in order to account for potential confounding of sex, ancestry and technical factors.
Blinding	This study used a case-control design, and therefore blinding is not applicable.

Reporting for specific materials, systems and methods

We require information from authors about some types of materials, experimental systems and methods used in many studies. Here, indicate whether each material, system or method listed is relevant to your study. If you are not sure if a list item applies to your research, read the appropriate section before selecting a response.

Materials & experimental systems

n/a	Involved in the study	
☒	☐	Antibodies
☒	☐	Eukaryotic cell lines
☒	☐	Palaeontology and archaeology
☒	☐	Animals and other organisms
☒	☐	Clinical data
☒	☐	Dual use research of concern
☒	☐	Plants

Methods

n/a	Involved in the study	
☒	☐	ChIP-seq
☒	☐	Flow cytometry
☒	☐	MRI-based neuroimaging

Plants

Seed stocks	*Report on the source of all seed stocks or other plant material used. If applicable, state the seed stock centre and catalogue number. If plant specimens were collected from the field, describe the collection location, date and sampling procedures.*
Novel plant genotypes	*Describe the methods by which all novel plant genotypes were produced. This includes those generated by transgenic approaches, gene editing, chemical/radiation-based mutagenesis and hybridization. For transgenic lines, describe the transformation method, the number of independent lines analyzed and the generation upon which experiments were performed. For gene-edited lines, describe the editor used, the endogenous sequence targeted for editing, the targeting guide RNA sequence (if applicable) and how the editor was applied.*
Authentication	*Describe any authentication procedures for each seed stock used or novel genotype generated. Describe any experiments used to assess the effect of a mutation and, where applicable, how potential secondary effects (e.g. second site T-DNA insertions, mosiacism, off-target gene editing) were examined.*
